# Reviewer acknowledgement

**DOI:** 10.1186/s13062-015-0038-9

**Published:** 2015-02-22

**Authors:** Eugene V Koonin, Laura F Landweber, David Lipman

**Affiliations:** National Center for Biotechnology, Information, National Library of Medicine, National Institutes of Health, Bethesda, MD USA; Department of Ecology and Evolutionary Biology, Princeton University, Princeton, NJ USA

## Abstract

The editors of *Biology Direct* would like to thank all the reviewers who have contributed to the journal in Volume 9 (2014).

**Anthony Almudevar**

United States of America

**Vivek Anantharaman**

United States of America

**Miguel A Andrade-Navarro**

Germany

**David Ardell**

United States of America

**Manju Bansal**

India

**Eric Bapteste**

France

**Juergen Brosius**

Germany

**Alexander Maxwell Burroughs**

United States of America

**Oliviero Carugo**

Austria

**Dragos Cretoiu**

Romania

**Thomas Dandekar**

Germany

**Valerian Dolja**

United States of America

**Mikhail Dozmorov**

United States of America

**Frank Eisenhaber**

Singapore

**Xiangdong Fang**

China

**Alistair Forrest**

Japan

**Patrick Forterre**

France

**Toni Gabaldon**

Spain

**Michael Galperin**

United States of America

**Nicolas Galtier**

France

**Zoltán Gáspári**

Hungary

**Rachel Gerstein**

United States of America

**Michael Gilchrist**

United States of America

**Uri Gophna**

Israel

**Dan Graur**

United States of America

**Neil Greenspan**

United States of America

**Nick Grishin**

United States of America

**Michael Gromiha**

India

**Yuriy Gusev**

United States of America

**Balazs Györffy**

Hungary

**Daniel Haft**

United States of America

**Wayne Hancock**

United States of America

**Leonid Hanin**

United States of America

**Martijn Huynen**

Netherlands

**Ollivier Hyrien**

United States of America

**Lakshminarayan Iyer**

United States of America

**Christine Jacob**

France

**Etienne Joly**

France

**I. King Jordan**

United States of America

**Marek Kimmel**

United States of America

**Lev Klebanov**

Czech Republic

**Rob Knight**

United States of America

**Fyodor Kondrashov**

Spain

**Eugene Koonin**

United States of America

**D. P. Kreil**

Austria

**Yang Kuang**

United States of America

**Vladimir Kuznetsov**

Singapore

**Doron Lancet**

Israel

**Nick Lane**

United Kingdom

**Qing Kay Li**

United States of America

**Han Liang**

United States of America

**Alexander Lobkovsky**

United States of America

**Purificacion Lopez-Garcia**

France

**Kira Makarova**

United States of America

**William Martin**

Germany

**Sergei Maslov**

United States of America

**James McInerney**

Ireland

**Armen Mulkidjanian**

Germany

**Robert Murphy**

United States of America

**Sandor Pongor**

Italy

**Anthony Poole**

New Zealand

**Vladimir Poroikov**

Russian Federation

**Shyam Prabhakar**

Singapore

**Mark Ragan**

Australia

**Nathalie Reuter**

Norway

**Isidore Rigoutsos**

United States of America

**Igor Rogozin**

United States of America

**Inaki Ruiz-Trillo**

Spain

**Andrey Rzhetsky**

United States of America

**Paul Schimmel**

United States of America

**Alexander Schleiffer**

Austria

**Christian Schönbach**

Japan

**Jeremy Selengut**

United States of America

**Fedor Severin**

Russian Federation

**George V. (Yura) Shpakovski**

Russian Federation

**Neil Smalheiser**

United States of America

**Berend Snel**

Netherlands

**Narayanaswamy Srinivasan**

India

**Giovanni Stracquadanio**

United States of America

**Manabu Sugai**

Japan

**Shamil Sunyaev**

United States of America

**Nektarios Tavernarakis**

Greece

**Lutz Walter**

Germany

**Christine Wells**

Australia

**Claus Wilke**

United States of America

**Limsoon Wong**

Singapore

**Soojin Yi**

United States of America

**Jun Yu**

China

**Weixiong Zhang**

United States of America

**Hong Zhou**

United States of America

**Igor Zhulin**

United States of America

**Piotr Zielenkiewicz**

Poland

